# The Complex QT/RR Relationship in Mice

**DOI:** 10.1038/srep25388

**Published:** 2016-05-03

**Authors:** Julien Roussel, Pascal Champeroux, Jérôme Roy, Sylvain Richard, Jérémy Fauconnier, Jean-Yves Le Guennec, Jérôme Thireau

**Affiliations:** 1 Inserm U1046, UMR CNRS 9214, Université de Montpellier, 34295 Montpellier France; 2 CERB, Chemin de Montifault, 18800 Baugy France

## Abstract

The QT interval reflects the time between the depolarization of ventricles until their repolarization and is usually used as a predictive marker for the occurrence of arrhythmias. This parameter varies with the heart rate, expressed as the RR interval (time between two successive ventricular depolarizations). To calculate the QT independently of the RR, correction formulae are currently used. In mice, the QT-RR relationship as such has never been studied in conscious animals, and correction formulas are mainly empirical. In the present paper we studied how QT varies when the RR changes physiologically (comparison of nocturnal and diurnal periods) or after dosing mice with tachycardic agents (norepinephrine or nitroprusside). Our results show that there is significant variability of QT and RR in a given condition, resulting in the need to average at least 200 consecutive complexes to accurately compare the QT. Even following this method, no obvious shortening of the QT was observed with increased heart rate, regardless of whether or not this change occurs abruptly. In conclusion, the relationship between QT and RR in mice is weak, which renders the use of correction formulae inappropriate and misleading in this species.

In 1906, Wilhem Einthoven published the use of the string galvanometer to record the ECG and described/named the P-Q-R-S-T waves^1^, for which he received the Nobel Prize for Physiology or Medicine in 1924. Subsequently, it has been shown that the QT interval, representing the duration of the ventricular depolarization of the action potential (AP), depends on the heart rate (HR). This is known as the QT-RR relationship, the RR interval being the period between two consecutive ventricular depolarizations. This physiological relationship presents great inter- and intra-individual variability, linked to the ortho- and para-sympathetic tones^2^.

An increase in contraction frequency, i.e. the HR, produces a positive inotropic effect whereby the force of the contraction increases and reaches a new steady state within a few beats. This phenomenon is named the “positive staircase”, Treppe or Bowditch effect, and affects the RR and QT intervals. This phenomenon is marked in species with a ventricular action potential characterized by a long plateau phase (as in humans, cats and pigs), which is the cellular expression of the QT^3^,^4^. For instance, in humans, an increase in pacing rate can accumulate I_KS_ in the open state and activate the Ca^2+^ dependent Cl- current (I_TO2_), resulting in a shortening of the action potential duration (APD)^5^,^6^,^7^,^8^.

Nevertheless, these electrophysiological determinants of the frequency-dependence of the APD differ between species. For example, in lagomorphs or rodents, with a short to very short refractory period, the dependence of the APD on frequency is less clear and could be contrary to the effects seen in humans^4^,^9^,^10^.

QT prolongation secondary to acquired or congenital heart disease or induced by pharmacological compounds is associated with an increased risk of arrhythmias^11^. Since the QT interval depends on the HR, a correction needs to be applied in order to render the QT interval independent of the RR interval, and to detect any APD changes independent of the HR. Bazett^12^ and Fridericia^13^, by analyzing a large number of ECGs, have empirically developed correction formulae that are the most used in humans. These formulas have subsequently been applied in animals, but it has been observed that the corrected QT (QTc) is overestimated at high HRs and underestimated at low HRs^14^. For this reason, many groups are attempting to establish an index more predictive of fatal arrhythmias, and more independent of HR correction formulae (see^14^ for review). Despite this, the QTc remains the most used index to evaluate the electrical properties of ventricles. Many formulae have been used to better fit specific QT-RR relationships (see^14^ for review), and a new formula, which can be adapted to different species or individuals, has recently been proposed^14^,^15^. Broadly, this approach, dedicated to pharmacological safety experiments, aims to linearize the QT-RR relationship, in order to characterize individual QT-RR relationships and determine whether compounds modify QT *per se*.

Mice are the most used animal species in research labs and preclinical studies. There are many strains of mice, wild-type or genetically engineered, with well-characterized cardiac electrophysiology^16^,^17^. The formulae used to correct the QT in mice are generally formulae developed previously for other species (mostly for humans), but their use sometimes yields quite surprising results, such as a QTc of 160 ms for a mean RR of 130 ms^18^. They are therefore empirically weighted to obtain a value of the same order as the raw QT. For example, the original Bazett’s formula developed for humans is 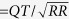, while the most used formula in mice is 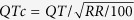, i.e. Bazett’s formula normalized to the mean cycle length of this species^19^,^20^,^21^. In addition to correction formulae, the method used to determine the QT over the ECG trace is also of interest. In many studies, QT is measured over small periods, with or without respect to the nychthemeral rhythm, and a correction formula applied subsequently, either by using beat-to-beat RR values, or a mean RR. In other studies, QTc is calculated using complex procedures. For example, Jeyaraj *et al.*^22^ calculated the QTc with a weighted HR approach where measurements were made every 2 hours over a 24-hour period. The mean hourly HR was calculated, and the QT interval measured only between two consecutive beats separated by an RR corresponding to the mean HR. Subsequently, Mitchell’s formula was applied to correct the QT interval hour by hour. Such different approaches make it difficult to compare the results of different studies. A dedicated study to better define the relationship between QT and RR in mice is needed in order to measure and correct the QT in a more accurate, logical and consistent manner.

To do this, we measured the ECG traces of 21 conscious C57Bl6 mice. Between 10000 and 30000 ECG complexes per mouse were analyzed at different periods (diurnal and nocturnal) under basal conditions, and after the injection of either nitroprusside or norepinephrine to evaluate intra-individual variability and the QT-RR relationship in mice.

Our results indicate that in mice, the QT interval depends on the RR interval to a negligible extent, leading us to conclude that correcting the QT, whatever the formula used, has no physiological value at best, and at worst, introduces errors.

## Results

**Figure 1:**
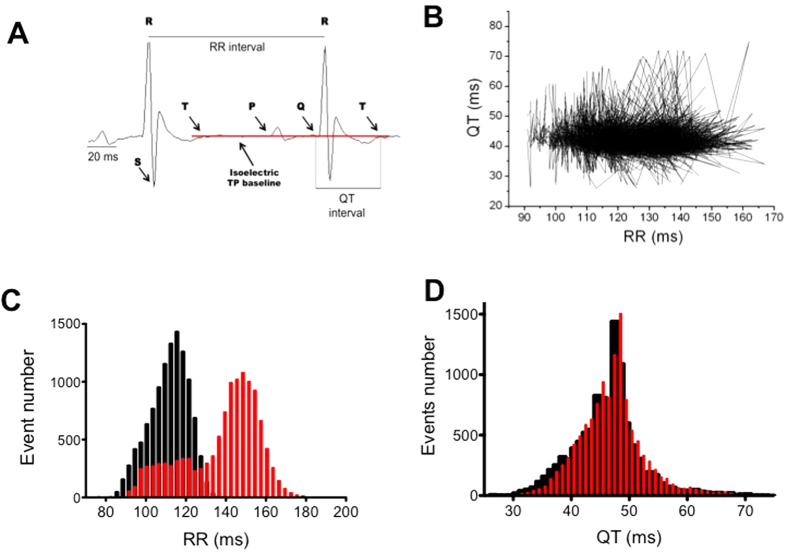
QT-RR relationship of a typical mouse. (**A**) Typical example of an ECG recording and ECG-wave placements. (**B**) QT-RR relationship recorded for 30 min during the nocturnal period. Pairs are linked by straight lines to emphasize the density of points in the middle of the cloud. (**C**) example of a frequency histogram of distribution of the RR interval during the nocturnal (black) and diurnal periods (red) measured during 30 min in the middle of each period. (**D**) QT frequency histogram of distribution from the same mouse than in B.

**Figure 2:**
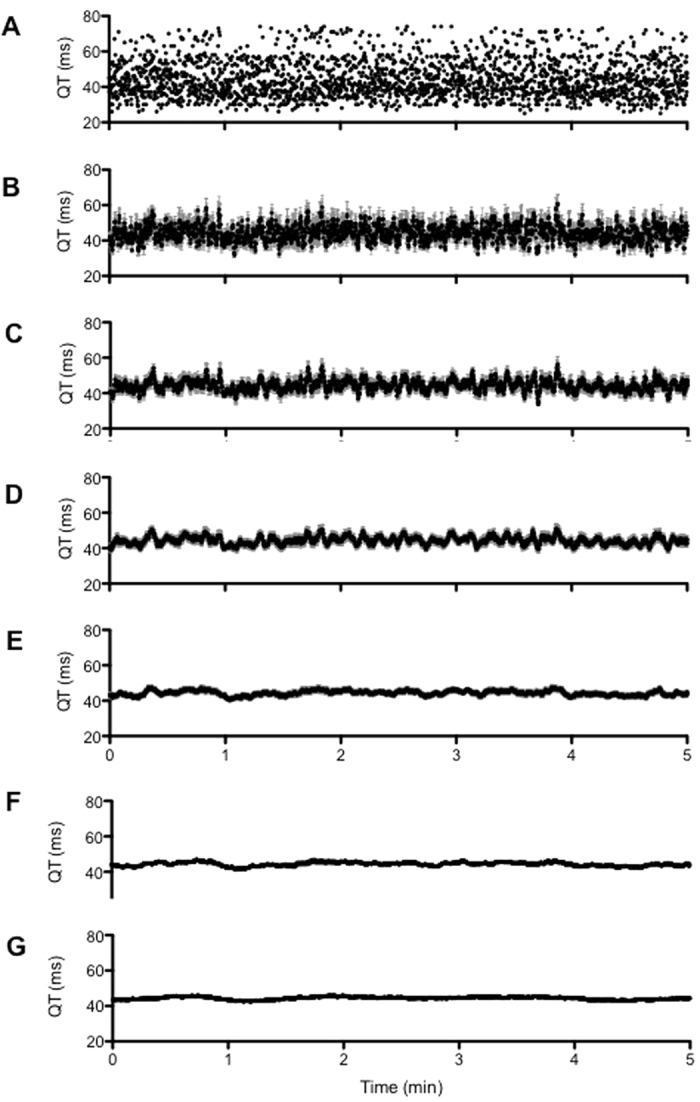
Beat-to-beat variation of the QT interval measured during a 5min time period and filtered thanks to a moving average of different subset sizes. (**A**) raw QT interval. (**B**) subset of 5 measurements. (**C**) subset of 11 measurements. (**D**) subset of 21 measurements. (**E**) subset of 51 measurements. (**F**) subset of 101 measurements. (**G**) subset of 201 measurements.

Beat-to-beat QT durations and RR intervals were measured during the diurnal and nocturnal periods from ECG signals in 11 male C57Bl/6 mice. Typical examples of the results are shown in Fig. 1. As presented in Fig. 1B, the QT and RR intervals varied considerably during the nocturnal period. An analysis of the distribution of the QT and RR intervals over 45–60 minutes during the middle of the nocturnal and diurnal periods indicated that the RR interval followed two Gaussian distributions (Fig. 1C). Although the QT exhibited a wide range of values (between 30 and 60 ms in Fig. 1B), it still followed a single normal distribution curve during both the nocturnal and diurnal periods (Fig. 1D). This suggests that QT is only weakly dependent on the nycthemeral cycle in mice, unlike the HR. One could therefore assume that, depending on the moment when QT intervals are measured, the values obtained could vary significantly independently of the RR. To determine how they vary and whether averaging successive QT values could bypass this problem, we measured all QT values in a 5-minute sequence of diurnal ECG recordings (n = 2205 QRS complexes) and compared raw values with those obtained with sliding averages of subsets of 5, 11, 21, 51, 101 and 201 consecutive complexes (Fig. 2). We observed that the extent of QT variation (maximum QT *minus* minimum QT) during the 5 min sequence was 49 ms without averaging, and this decreased to 29, 22.5, 12, 7, 5.6 and 3.4 ms when averaging 11, 21, 51, 101 and 201 consecutive complexes respectively. Thus, in the rest of the present manuscript, QT intervals were calculated by averaging at least 200 complexes.

**Figure 3:**
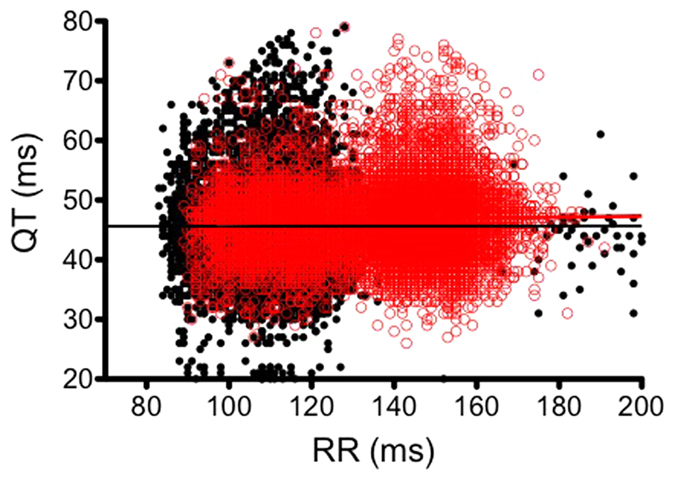
QT-RR relationship recorded over 30min during the nocturnal (black) and the diurnal (red) periods (see Method section). Straight lines indicate the linear regression line calculated during both periods.

Figure 1B shows that the data points formed a cloud with no obvious relationship between QT and RR, and where QT varies from ≈30 to 60 ms and RR varies from 90 to 160 ms. In addition, the points above and below the cloud are erratic and are not consecutive to abrupt RR changes. Since it was difficult to establish a clear relationship between QT and RR during nocturnal period, we analyzed the ECG during the diurnal period, when the HR is expected to slow down. We thus plotted QT as a function of RR during both the nocturnal and diurnal periods (example in Fig. 3). The relationship between QT and RR was flat (r^2^ = 0.07) in the example given and could even be negative for some mice (Table 1). Of the 22 QT-RR series analyzed, 4 relationships between QT and RR were not significant, 6 were negative and significant and 12 were positive and significant.

**Figure 4:**
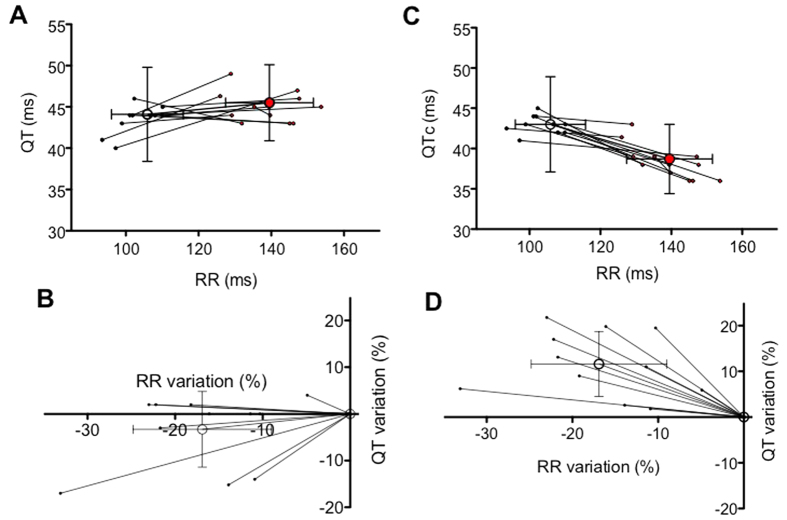
Influence of the nocturnal and diurnal periods on mean QT-RR relationships of eleven mice. Open circles correspond to the mean values obtained from different animals and the standard deviation in RR and QT are given and the filled circles the individual measurement. (**A**) Individuals and mean QT-RR relationships during the diurnal (black) and nocturnal periods (red points). (**B**) Variations in QT and RR expressed in percentage of the respective values obtained during the diurnal period. (**C**) Individuals and mean QTc-RR relationships during the diurnal and nocturnal periods. The QTc has been calculated according to equation (1). (**D**) Variations in QTc and RR expressed in percentage of the respective values calculated during the diurnal period.

When we compared the mean changes in QT and RR between nocturnal and diurnal periods in 11 mice, we observed that, even if there was a tendency for QT to decrease with decreased RR, this change was not statistically significant and the inter-individual variability was rather large (Fig. 4A). Thus, to decrease this variability, we plotted the variation of QT, expressed as a percentage, during the diurnal as well as during the nocturnal period (Fig. 4B). Here again we could observe that there was a slight, but not significant, tendency for the QT to decrease with decreased RR (also expressed as a percentage of median RR during the diurnal period).

To evaluate the consequences of using correction formulae such as equation (1), which is the most used in the literature^19^, we plotted the same data as shown in Fig. 4A,B, but using QTc instead of the raw QT. As shown in Fig. 4C,D, the mean variations in QTc became significant when moving from the diurnal to the nocturnal period. Moreover, and unexpectedly, while RR intervals were longer, QTc values were inversely shorter.

**Figure 5:**
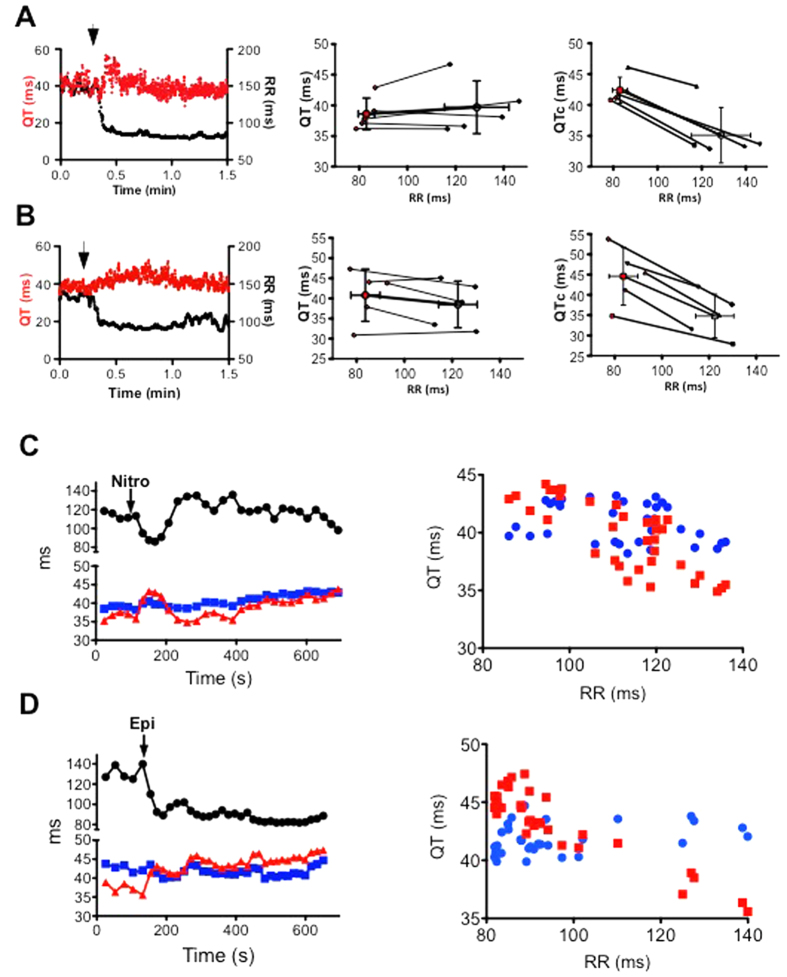
Influence of abrupt changes in cardiac frequency on QT interval and RR intervals in 5 mice before (black) and after (red points) IP injection of norepinephrine (**A**) or nitroprusside (**B**). Open circles give the mean RR and QT ± SD and filled circles the individual measurements. Left, typical evolution of QT (red) and RR (black) intervals just before and in the minute following the injection; middle, raw QT-RR relationship; right, QTc-RR relationship. Evolution of the QT and QTc intervals according to the RR intervals over-time. QT (blue points), QTc (red points) and RR intervals (black points) were measured 5 minutes before and 15 minutes after injection of nitroprusside (**C**) or Epinephrine (**D**). QTc were calculated according to Mitchell’s formula.

It thus appears that physiological changes in the RR interval due to the nycthemeral cycle are not associated with significant changes in QT. However, this does not rule out the possibility that a QT-RR relationship that is not steep still exists. To test this possibility, we evaluated the impact on the QT interval of rapid and more pronounced changes in the RR interval induced by norepinephrine (NE) or nitroprusside (Fig. 5).

When mice were challenged with NE, there was a rapid and large decrease of the RR interval, but QT intervals did not vary (Fig. 5A). Since NE can directly interfere with the regulation of cardiac electrical activity, we used another tachycardic agent, the vasodilator nitroprusside, which does not have a direct cardiac effect (Fig. 5B). As observed with NE, changes in the RR were not associated with changes in QT in the minute following the injection. To verify that this poor dependence of the QT on the RR was not due to the low adaptive sensitivity of the QT to the RR, we measured the RR, QT and QTc intervals 15 minutes after drug administration (Fig. 5C,D). In the case of both drugs, correcting the QT with Mitchell’s formula introduced an apparent dependence of the QTc on the RR. Again, this dependence was contrary to the expected adaptation of the QT to the stimulation frequency: QTc increased as the HR increased.

## Discussion

Hundreds of articles have used the QTc value to assess repolarization time in mice and investigate the effects of environmental changes (due to drugs or disease) on ventricular repolarization. Here, we asked whether there was a QT/RR relationship in mice using two approaches: by investigating the well-known physiological changes in the RR interval during nycthemeral cycles, and by the pharmacological alteration of the HR. Our study principally shows that the QT-RR relationship in mice is so tenuous that, whatever the correction formula chosen, it can only introduce a bias in any calculation that depends solely on changes in the RR interval. Speerschneider & Thomsen have already described the lack of dependence of the QT on the RR in anesthetized mice, and shown that correction formulae erroneously introduce such dependence^23^. However, they did not record the ECG in conscious mice, which have a faster HR.

In contrast to other species such as humans, pigs, dogs or guinea-pigs, in which there is a clear QT-RR relationship that needs to be determined for each individual^14^, this relationship is not obvious in mice. Thus, precautions need to be taken when interpreting ventricular depolarization time in mice from the raw QT and/or following QT correction. We also uncovered great inter- and intra-individual variability in mice, indicating that a large number of animals is required when analyzing QT parameters, in order for the data to be reliable.

The lack of a QT-RR relationship can be linked to the peculiarity of ventricular repolarization in mice, which starts before the activation of another cardiac territory^24^,^25^,^26^. Moreover, theoretically, the ventricular APD depends on the duration of the preceding diastolic period in a relationship termed “electrical restitution”. In humans, the APD becomes shorter with decreasing cycle lengths and thus with decreasing diastolic periods (a phenomenon also named “cardiac memory”). This mechanism allows for an increase in the duration of excitation-contraction coupling during bradycardia (long cycle lengths) while favoring diastolic time for coronary perfusion and ventricular filling when the HR increases (reduced cycle lengths). Measuring the electrical restitution of the APD is thus considered to be better than only recording the APD at a fixed cycle length to assess the rate-dependence of the repolarization parameter, because it better reflects the adaptation of the repolarization time to physiological variations of the HR. Unlike what happens in humans^27^, the APD restitution curve for late repolarization (APD_90_) in mice is nearly flat, as elegantly shown by Knollmann and collaborators^28^. The relative APD restitution curve for early repolarization (APD_30_) even shortens when the stimulation interval lengthens. Accordingly, Nuyens *et al.*^29^ have shown that a rapid change in stimulation pacing does not affect the APD_90_ in wild-type mice. Interestingly, in the latter two studies, “steady-state” APD_90_ measurements i.e. those measured at a fixed cycle length, exhibited stimulation rate-dependence, although this was less important than in others mammals such as dogs and humans. This phenomenon could be explained by the fact that the ionic currents determining the duration of action potentials are different in mice and humans. Potassium currents in mice do not accumulate upon repetitive stimuli but instead slowly inactivate, which explains why their action potential only moderately shortens at rapid stimulation rates^30^.

Another explanation could also be related to the difficulty involved in accurately measuring the T wave in mice^23^. Indeed, in larger animals in which the analysis of the QT is complex, electrodes have to be placed directly on the cardiac surface to be able to measure variations of the QTc in the millisecond range^31^. This aspect is important since the QT variations observed in mice are also in this range, suggesting strongly that it would be very difficult to define a QT-RR relationship using the usual methods. More work to improve methodological approaches and T-wave detection in mice and to standardize ECG-lead placements is still needed to reduce potential analytical errors during T-wave determination. In the same way, choosing an averaged RR interval to correct the averaged QT in mice, or picking a given QT instead of another based on the RR interval, could introduce a major bias in the analysis by erroneously creating a relationship between two weakly dependent variables.

Regarding our data, we propose that the lack of a QT-RR relationship is of physiological origin, since QT values were not significantly different between the diurnal and nocturnal periods, when RR values were very different. Small rodents have a rather low range of RR intervals compared to larger animals (see^14^). For example, Kaese & Verheule^16^ have reported that mice have the capacity to increase their HR by only 30–40%, compared to 300% in humans. In our study, similar results were obtained with pharmacological treatment. Under such conditions, variations in QT must be rather small, if they exist, due to the fast HR. The mouse action potential, in contrast to that of larger mammals, is short and probably acts more as a trigger for contraction, with less mechanical feedback, than in mammals that display a plateau phase in their action potential^32^.

Another important finding of the present study is the intra-individual variability of the QT^16^,^33^,^34^. Indeed, for a given experiment, mean QT values are often calculated by averaging a few complexes at different time points. However, any change in QT duration might be consecutive to the physiological variation of the QT rather than to the condition tested. This information would be lost with the use of correction formulae.

In conclusion, we have demonstrated in the present study that there is no obvious QT-RR relationship in mice, and that the use of correction formulae such as those adapted from Bazett’s formula would merely introduce errors.

## Methods

### Ethics

Ten-week-old male C57Bl/6 mice (Janvier, France) were used in this study (11 for diurnal/nocturnal study and 10 for pharmacological testing). All procedures conformed to European Parliament Directive 2010/63/EU and the 22 September 2010 Council on the protection of animals, and were approved by the institutional animal research committee (Departmental Directorate of protecting populations and animal health (ethics for animal welfare and environmental protection, N° A 34–485) and by our Ethics committee for animal experiments, Languedoc Roussillon, N° CE-LR-0714).

### QT specification and determination in mice

The duration of QRS-T complexes reflects the duration of ventricular depolarization and repolarization. Due to specific electrophysiological properties at cardiomyocytes level with predominant I_TO_ current, the murine action potential lacks plateau phase. Repolarization in mice starts when another cardiac territory is not yet activated^24^,^25^,^26^. Combined with the short RR interval (≈100 ms), the ST segment and the T-waves are difficult to identify from ECG trace, leaving some authors to suggest that T wave in mice does not exist^24^,^35^,^36^,^37^. Nevertheless the same concepts than in human ECG are actually used for the interpretation of theses rodent ECGs. Thus, several groups have tempted to empirically define the best estimation methods to define QT duration (as well reviewed in^38^).

To date, the most used method to define the QT duration in mice is to consider the QT interval between the first deviation from an isoelectric PR interval until the return of the ventricular repolarisation to the isoelectric TP baseline, acquired by telemetry in vigil mice. This method included in the measure the low-amplitude portion of the T-wave and allows a complete ventricular repolarization of ventricles, as confirmed by a simultaneous record of monophasic ventricular action potential with ECG trace in mice^39^. By this method the QT interval found in the literature ranges from 40 to 60 ms in unsedated mice with an RR interval comprised between 85 to 115 ms.

### ECG recording and analysis

ECG were recorded using implantable TA10ETA-F10 ECG transmitters (Data Sciences International). Mice were instrumented under general gaseous anesthesia (2–2.5% isoflurane/O2, Aerrane). The transmitter device was inserted subcutaneously along the back, and the two ECG electrodes were placed subcutaneously in the region of the right shoulder (negative lead) and toward the lower left chest (positive lead) to approximate lead II of the Einthoven surface ECG Measurements were performed 2 weeks after total recovery from surgery, which allows retrieving complete HR variability i.e. a normal regulation of heart rhythm by autonomic nervous system^40^. Mice were then monitored with ECG recordings in their home cage with a signal transmitter-receiver (RPC-1) connected to a data acquisition system (Physiotel, Ponemah software, Data Sciences International). ECG were collected continuously over 24 h at a sampling rate of 2000 Hz in a dedicated room with respect to diurnal-nocturnal variations (12h/12h cycle), controlled hygrometry and temperature. Mice have access *ad libitum* to water and feed. Continuous 30 minutes digital recordings obtained during nocturnal period, i.e. during activity phase, were analyzed off line with Ponemah software, 3 hours after the turn on or up of the lights. At least 10.000 PQRST cycles, exclusively during sinus rhythm, were analyzed per condition. QRS complexes from ventricular extrasystoles, premature ventricular contractions or atrio-ventricular blocks were systematically rejected for the analyses. The good identification of ECG waves by the software and the good positioning of the cursors were validated “beat to beat” or modified by operator if needed (<5%). QT duration was defined as the interval between the first deviation from the isoelectric PR interval (Q wave) until the return of the ventricular repolarization to the isoelectric TP baseline from lead II ECGs. If one doubt existed due to the lack of stable isoelectrical line, an abnormal morphology of PQRST waveform, or chaotic electrical noise due to movement artifact, the PQRST sequence was not included in the analysis until next valuable complexes. When correction formula was applied, each QT was corrected to its own RR interval measured between two consecutive PQRST complexes, no average of RR intervals was performed to correct QT interval.

To test the strength of QT measurement and QT correction over a same mice, QT intervals were also measured over at least 200 consecutives QRS complexes randomly picked each 20 minutes over one hour (according to theirs good detection as mentioned earlier), and QT was corrected with the formula of Mitchell^19^:


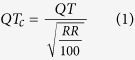


### Pharmacology

To study the relationship between the QT interval and cardiac frequency, we used two different approaches. The first one consisted in the measurement and analysis of RR and QT during nocturnal periods (high activity periods in mice) and during diurnal periods (periods of rest for mice). Beside these physiological changes in cardiac frequency, we also injected intraperitoneously 1 mg.kg^−1^ epinephrine or 1 mg.kg^−1^ nitroprusside in diurnal periods to trigger tachycardia by two different pathways. With epinephrine, the tachycardia is consecutive to the β-adrenergic activation at the sinusal level while with nitroprusside, it is consecutive to the hypotension induced by the drug (baroreflex activation). Measurements were performed five minutes before and fifteen minutes after dosing.

Epinephrine and nitroprusside were obtained from Sigma-Aldrich (Grenoble, france).

### Statistics

Analysis and graphs were produced thanks to Prism®. Pearson’s r correlation was determined for each QT-RR plots during the diurnal and the nocturnal periods. To compare the mean QT and RR during the diurnal or nocturnal periods, a paired student t-test was used. In all cases, p < 0.05 was considered as significant.

## Additional Information

**How to cite this article**: Roussel, J. *et al.* The Complex QT/RR Relationship in Mice. *Sci. Rep.* **6**, 25388; doi: 10.1038/srep25388 (2016).

## Figures and Tables

**Table 1:** Parameters of linear regression analysis of the QT-RR relationship of the eleven mice studied in diurnal and nocturnal periods.

Mouse/Period	Slope	Diff	r	Stats
1 diurnal nocturnal	0.0006	46.2	0.002	ns
	−0.015	42.2	0.037	*
2 diurnal nocturnal	−0.014	49.3	0.032	*
	−0.0057	41.3	0.013	ns
3 diurnal nocturnal	0.0227	40.2	0.089	*
	0.023	33.0	0.045	*
4 diurnal nocturnal	0.0126	44.8	0.044	*
	−0.0023	45.9	0.002	ns
5 diurnal nocturnal	0.0082	44.5	0.020	*
	0.0455	39.1	0.067	*
6 diurnal nocturnal	−0.1518	58.8	0.610	*
	−0.1550	60.8	0.637	*
7 diurnal nocturnal	0.0268	40.0	0.093	*
	0.0552	40.4	0.160	*
8 diurnal nocturnal	0.0125	42.7	0.054	*
	0.0306	40.2	0.143	*
9 diurnal nocturnal	−0.0012	43.8	0.003	ns
	0.1548	27.2	0.419	*
10 diurnal nocturnal	−0.0173	47.7	0.089	*
	−0.0042	41.5	0.060	*
11 diurnal nocturnal	−0.0043	49.9	0.020	*
	0.0069	44.0	0.036	*

The linear regression being calculated being: QTr = slope *RR + Diff. r is the Pearson correlation parameter and stats indicates the significance of the correlation. *p < 0.05. ns: non significant.

## References

[b1] Einthoven W. Le télécardiogramme. Arch Int de Physiol 4, 132–164 (1906).

[b2] Champéroux P. *et al.* Short term variability in QT interval and ventricular arrhythmias induced by dofetilide are dependent on high-frequency autonomic oscillations. Br J Pharm 172 **(11)**, 2878–2891 (2015). 10.1111/bph.13093 PMC4439882 25625756

[b3] Bowditch H. P. Über die eigenthümlichkeiten der reizbarkeit welche die muskelfasern des herzens zeigen. Arb Physio Aust 6, 139–176 (1871).

[b4] Carmeliet E. Intracellular Ca2+ concentration and rate adaptation of the cardiac action potential. Cell Calcium 35, 557–573 (2004). 15110146 10.1016/j.ceca.2004.01.010

[b5] Endoh M. Force–frequency relationship in intact mammalian ventricular myocardium: physiological and pathophysiological relevance. Eur J Pharmacol 500, 73–86 (2004). 15464022 10.1016/j.ejphar.2004.07.013

[b6] Li G., Feng J., Wang Z., Fermini B. & Nattel S. Comparative mechanisms of 4-aminopyridine-resistant Ito in human and rabbit atrial myocytes. American Journal of Physiology - Heart and Circulatory Physiology 269 **(2)**, H463–H472 (1995). 10.1152/ajpheart.1995.269.2.H463 7653610

[b7] Wang Z. *et al.* Potential molecular basis of different physiological properties of the transient outward K+ current in rabbit and human atrial myocytes. Circ Res 84 **(5)**, 551–61 (1999). 10082477 10.1161/01.res.84.5.551

[b8] Ravens U. & Wettwer E. Electrophysiological aspects of changes in heart rate. Basic Res Cardiol 93 suppl 1, 60–65 (1998). 9833132 10.1007/s003950050220

[b9] Fauconnier J., Bedut S., Le Guennec J.-Y., Babuty D. & Richard S. Ca2+ current-mediated regulation of action potential by pacing rate in rat ventricular myocytes. Cardiovasc Res 57 **(3)**, 670–680 (2003). 12618229 10.1016/s0008-6363(02)00731-9

[b10] Sallé L., Kharche S., Zhang H. & Brette F. Mechanisms underlying adaptation of action potential duration by pacing rate in rat myocytes. Prog Biophys Mol Biol 96 **(1–3)**, 305–320 (2010). 17869329 10.1016/j.pbiomolbio.2007.07.008

[b11] Vandenberg J. I. *et al.* hERG K(+) channels: structure, function, and clinical significance. Physiol Rev 92 **(3)**, 1393–1478 (2012). 22988594 10.1152/physrev.00036.2011

[b12] Bazett H. An analysis of the time-relations of electrocardiograms. Heart 7, 353–370 (1920).

[b13] Fridericia L. Die systolendauer im elektrokardiogramm bei normal menschen und bei herzkranken. Acta Med Scand 53, 469–486 (1920).

[b14] Holzgrefe H. *et al.* Preclinical QT safety assessment: cross-species comparisons and human translation from an industry consortium. J Pharmacol Toxicol Methods 69 **(1)**, 61–101 (2014). 23689033 10.1016/j.vascn.2013.05.004

[b15] Holzgrefe H. *et al.* Novel probabilistic method for precisely correcting the QT interval for heart rate in telemetered dogs and cynomolgus monkeys. J Pharmacol Toxicol Methods 55 **(2)**, 159–175 (2007). 16857392 10.1016/j.vascn.2006.05.007

[b16] Kaese S. & Verheule S. Cardiac electrophysiology in mice: a matter of size. Front Physiol 3, 345 (2012). 22973235 10.3389/fphys.2012.00345 PMC3433738

[b17] Derangeon M., Montnach J., Baro I. & Charpentier F. Mouse model of SCN5A-related cardiac arrhythmias. Front Physiol 3, 210 (2012). 22737129 10.3389/fphys.2012.00210 PMC3381239

[b18] Decher N. *et al.* Knock-out of the potassium channel TASK-1 leads to a prolonged QT interval and a disturbed QRS complex. Cell Physiol Biochem 28, 77–86 (2011). 21865850 10.1159/000331715

[b19] Mitchell G. F., Jeron A. & Koren G. Measurement of heart rate and QT interval in conscious mouse. Am J Physiol Heart Circ Physiol 274, H747–H751 (1998). 10.1152/ajpheart.1998.274.3.H747 9530184

[b20] Gélinas R. *et al.* Prolonged QT interval and lipid alterations beyond β-oxidation in very long-chain acy-CoA dehydrogenase null mouse hearts. Am J Physiol Heart Circ Physiol 301, H813-HH823 (2011). 10.1152/ajpheart.01275.2010 PMC3191095 21685264

[b21] Huang H. *et al.* Diet-induced obesity causes long QT and reduces transcription of voltage-gated potassium channels. J Mol Cell Cardiol 59, 151–158 (2013). 23517696 10.1016/j.yjmcc.2013.03.007 PMC3647001

[b22] Jeyaraj D. *et al.* Circadian rhythms govern cardiac repolarization and arrhythmogenesis. Nature 483, 96–99 (2012). 22367544 10.1038/nature10852 PMC3297978

[b23] Speerschneider T. & Thomsen M. B. Physiology and analysis of the electrocardiographic T wave in mice. Acta Physiol (Oxf) 209 **(4)**, 262–271 (2013). 24119104 10.1111/apha.12172

[b24] Richards A. G., Simonson E. & Visscher M. G. Electrocardiogram and phonogram of adult and newborn mice in normal conditions and under the effects of cooling, hypoxia and potassium. Am J Physiol 174, 293–298 (1953). 13080449 10.1152/ajplegacy.1953.174.2.293

[b25] London B. Cardiac arrhythmia: from (transgenic) mice to men. J cardiovasc electrophysiol 12, 1089–1091 (2001). 11573703 10.1046/j.1540-8167.2001.01089.x

[b26] Boukens B. J. Early repolarization in mice causes overestimation of ventricular activation time by the QRS duration. Cardiovasc Res 97, 182–191 (2013). 22997159 10.1093/cvr/cvs299

[b27] Franz M., Swerdlow C., Liem L. & Schaefer J. Cycle length dependence of human action potential duration *in vivo*. Effects of single extrastimuli, sudden sustained rate acceleration and deceleration, and different steady-state frequencies. J Clin Invest 82 **(3)**, 972–979 (1988). 3417875 10.1172/JCI113706 PMC303610

[b28] Knollmann B. Action potential characterization in intact mouse heart: length dependence and electrical restitution, Am J Physiol Heart Circ Physiol 292, H614–H621 (2007). 16963611 10.1152/ajpheart.01085.2005

[b29] Nuyens D. *et al.* Abrupt rate accelerations or premature beats cause life-threatening arrhythmias in mice with long-QT3 syndrome. Nat Med 7 **(9)**, 1021–1027 (2001). 11533705 10.1038/nm0901-1021

[b30] Nerbonne J. M. Molecular basis of functional voltage-gated K + channel diversity in the mammalian myocardium. J Physiol 525 **(2)**, 285–298 (2000). 10835033 10.1111/j.1469-7793.2000.t01-1-00285.x PMC2269952

[b31] Champéroux P. *et al.* Calculation of QT shift in non clinical safety pharmacology studies. J Pharmacol Toxicol Methods 59 **(2)**, 73–85 (2009). 19135537 10.1016/j.vascn.2008.11.001

[b32] Lab M. J. Mechanoelectric feedback (transduction) in heart: concepts and implications. Cardiovasc. Res 32 **(1)**, 3–14 (1996). 8776398

[b33] Scicluna B. P. *et al.* Quantitative loci for electrocardiographic parameters and arrhythmia in the mouse. J Mol Cell Cardiol 50, 380–389 (2011). 20854825 10.1016/j.yjmcc.2010.09.009

[b34] Petric S. *et al.* *In vivo* electrophysiological characterization of TASK-1 deficient mice. Cell Physiol Biochem 30, 523–537 (2012). 22813543 10.1159/000341435

[b35] Agduhr E. & Stenström N. The appearance of the electrocardiogram in heart lesions roduced by cod liver oil treatment. Acta Paediatrica 10 **(1–2)**, 271–280 (1930).

[b36] Lombard E. A. Electrocardiograms of Small Mammals. Am J Physiol 171, 189–193 (1952). 12985980 10.1152/ajplegacy.1952.171.1.189

[b37] Liu G. *et al.* *In vivo* temporal and spatial distribution of depolarization and repolarization and the illusive murine T wave. J Physiol 555 **(1)**, 267–79 (2004). 14634200 10.1113/jphysiol.2003.054064 PMC1664824

[b38] Zhang Y., Wu J., King J. H., Huang C. L. & Fraser J. A. Measurement and interpretation of electrocardiographic QT intervals in murine hearts. Am J Physiol Heart Circ Physiol 306 **(11)**, H1553-H1557 (2014). 10.1152/ajpheart.00459.2013 PMC4042200 24705556

[b39] Danik S. *et al.* Correlation of repolarization of ventricular monophasic action potential with ECG in the murine heart. Am J Physiol Heart Circ Physiol 283 **(1)**, H372–381 (2002). 12063311 10.1152/ajpheart.01091.2001

[b40] Thireau J., Zhang B. L., Poisson D. & Babuty D. Heart rate variability in mice: a theoretical and practical guide. Exp Physiol 93 **(1)**, 83–94 (2008). 17911354 10.1113/expphysiol.2007.040733

